# Surgical Resection plus Intraoperative Radiofrequency Ablation versus Chemoembolization for the Treatment of Intermediate-Stage (BCLC B) Hepatocellular Carcinoma with Preserved Liver Function: A Propensity Score-Matched Analysis

**DOI:** 10.3390/cancers14102440

**Published:** 2022-05-15

**Authors:** Gun Ha Kim, Jin Hyoung Kim, Heung Kyu Ko, Hee Ho Chu, Seong Ho Kim, Ji Hoon Shin, Dong Il Gwon, Gi-Young Ko, Hyun-Ki Yoon, Ki-Hun Kim, Ju Hyun Shim, Nayoung Kim

**Affiliations:** 1 Department of Radiology, Asan Medical Center, University of Ulsan College of Medicine, 88 Olympic-ro 43-gil, Songpa-gu, Seoul 05505, Korea; kimgh.rad@amc.seoul.kr (G.H.K.); angiochu@amc.seoul.kr (H.H.C.); evazzang23@amc.seoul.kr (S.H.K.); jhshin@amc.seoul.kr (J.H.S.); radgwon@amc.seoul.kr (D.I.G.); kogy@amc.seoul.kr (G.-Y.K.); hkyoon@amc.seoul.kr (H.-K.Y.); 2 Department of Surgery, Asan Medical Center, University of Ulsan College of Medicine, 88 Olympic-ro 43-gil, Songpa-gu, Seoul 05505, Korea; khkim620@amc.seoul.kr; 3 Department of Gastroenterology, Asan Medical Center, University of Ulsan College of Medicine, 88 Olympic-ro 43-gil, Songpa-gu, Seoul 05505, Korea; s5854@amc.seoul.kr; 4 Department of Clinical Epidemiology and Biostatistics, Asan Medical Center, University of Ulsan College of Medicine, 88 Olympic-ro 43-gil, Songpa-gu, Seoul 05505, Korea; nyny0803@amc.seoul.kr

**Keywords:** hepatocellular carcinoma, surgery, radiofrequency ablation, transarterial chemoembolization

## Abstract

**Simple Summary:**

Surgical resection plus intraoperative radiofrequency ablation showed better survival outcomes than transarterial chemoembolization in selected patients with intermediate-stage hepatocellular carcinoma and Child–Pugh class A liver function. These findings suggest that surgical resection plus intraoperative radiofrequency ablation may provide an opportunity for curative treatment to selected patients deemed eligible only for palliative treatment.

**Abstract:**

The purpose of this study was to compare the efficacy and safety of surgical resection (SR) plus intraoperative radiofrequency ablation (IORFA) with transarterial chemoembolization (TACE) in patients with intermediate-stage HCC and Child–Pugh class A liver function. Treatment-naïve patients who received SR plus IORFA (*n* = 104) or TACE (*n* = 513) were retrospectively evaluated. Patients were subjected to a maximum 1:3 propensity score matching (PSM), yielding 95 patients who underwent SR plus IORFA and 252 who underwent TACE. Evaluation of the entire study population showed that progression-free survival (PFS) and overall survival (OS) were significantly better in the SR plus IORFA than in the TACE group. After PSM, the median PFS (18.4 vs. 15.3 months) and OS (88.6 vs. 56.2 months) were significantly longer, and OS rate significantly higher (HR: 0.65, *p* = 0.026), in the SR plus IORFA group than in the TACE group. Stratified Cox regression analysis and doubly robust estimation revealed that treatment type was significantly associated with both OS and PFS. Rates of major complications were similar in the SR plus IORFA and TACE groups. In conclusion, SR plus IORFA showed better survival outcomes than TACE. SR plus IORFA may provide curative treatment to patients with intermediate-stage HCC with ≤4 tumors and Child–Pugh class A.

## 1. Introduction

Hepatocellular carcinoma (HCC) is the fifth most frequent malignancy and the third leading cause of cancer-related deaths worldwide [1]. The Barcelona Clinic Liver Cancer (BCLC) staging system and the European Association for the Study of the Liver guidelines recommend transcatheter arterial chemoembolization (TACE) as first-line therapy for patients with intermediate-stage (BCLC B) HCC [1]. The development of super-selective techniques and TACE devices has gradually improved patient survival, with selected patients having a median overall survival (OS) of up to 40 months [1]. However, the long-term survival outcomes of patients with BCLC B HCC who underwent TACE do not appear to be completely satisfactory, with several studies showing that surgical resection (SR) provided better survival outcomes than TACE in selected patients [2,3].

SR is considered a curative treatment option for patients with early stage HCC, but its use is restricted by factors such as insufficient future liver remnant (FLR), unfavorable tumor location, and multifocal tumors for patients with BCLC B HCC [4]. However, many institutions have offered more aggressive treatment whenever possible in a wider group of patients than proposed in the BCLC algorithm to achieve better survival outcomes [5]. Radiofrequency ablation (RFA) is an alternative/substitute treatment modality for early stage HCC, with survival outcomes comparable to those of SR alone [6,7]. SR plus intraoperative RFA (IORFA) involves the resection of surgically favorable superficial or multifocal HCCs confined to a single lobe, accompanied by IORFA of small but surgically unfavorable and unresectable HCCs located deep in the liver or near major vasculature, thus maximizing FLR. To date, SR plus IORFA has been shown to be safe and effective for various multifocal primary and secondary liver malignancies, including colorectal cancer metastases to the liver [8,9,10]. This combination may be a curative treatment option for eligible patients with BCLC B HCC. Indeed, several studies have shown that SR plus IORFA is a safe and effective treatment option for patients with multifocal HCC. Few studies, however, have compared the efficacy and safety of SR plus IORFA with those of TACE, with those studies limited by their small sample sizes [11,12,13]. The present study therefore compared the efficacy and safety of SR plus IORFA with TACE in patients with intermediate-stage (BCLC B) HCC.

## 2. Materials and Methods

### 2.1. Study Patients

This study was approved by the institutional review board, which waived the need for patient informed consent due to the retrospective design of the study. HCC was diagnosed according to the guidelines of the American Association for the Study of Liver Diseases (AASLD) or the European Association for the Study of the Liver (EASL) [1,14].

In our institution, the indications for SR plus IORFA in patients with BCLC B HCC and preserved liver function include (a) a completely resectable main HCC; (b) ≤3 remnant HCC nodules, each ≤3 cm in maximum diameter; and (c) if SR alone had a high risk of insufficient FLR or was impossible due to unfavorable tumor locations, as determined by a multidisciplinary team. To increase the comparability of SR plus IORFA with TACE, treatment-naïve patients with HCC were included if they (a) underwent SR plus IORFA or TACE as first-line treatment between January 2009 and December 2021; (b) were diagnosed with intermediate (BCLC B)-stage HCC and had ≤4 nodules; (c) had Child–Pugh class A liver function; and (d) had an Eastern Cooperative Oncology Group performance status of 0. Patients were excluded if they had (a) major vascular invasion or extrahepatic metastasis, (b) Child–Pugh class B or C liver function, or (c) previous or current malignancy other than HCC. Patients were also excluded if they were lost to follow-up after SR plus IORFA or TACE, and patients in the TACE group were excluded if they had undergone preoperative TACE. The physicians explained the treatment options to all eligible patients, with the treatment modality for each patient chosen after considering the preferences of the physician and patient, as well as the cost.

### 2.2. Surgical Resection plus Intraoperative Radiofrequency Ablation

Intraoperative US (Avius, Hitachi Aloka, Tokyo, Japan) was performed during surgery to identify HCCs and characterize their proximities to adjacent vascular structure. The decision to perform anatomic resection was dependent on the location of HCCs, as well as their proximity to adjacent vessels and bile ducts. The extent of SR was determined on the basis of the estimated hepatic functional reserve, as determined by the sizes of HCCs, preoperative liver biochemistry, and predicted FLR after SR.

IORFA was performed by an interventional radiologist with 20 years of experience. Before SR of the main HCCs, intraoperative US was performed to evaluate other HCCs compatible with preoperative computed tomography (CT) and/or magnetic resonance imaging (MRI). Because distinct visualization of the HCCs may be difficult due to the coarse echogenicity of cirrhotic liver, HCCs were differentiated by Sonazoid (GE Healthcare, Oslo, Norway). After SR of the main HCC, a single 17-G internally cooled electrode (Proteus, STARmed, Goyang, Korea) was inserted into the center of each remaining HCC under US guidance with a 7 MHz convex probe. IORFA was performed for 12 min using a 200 W generator (Viva RF system, STARmed, Goyang, Korea) in automatic impedance mode. The endpoint of IORFA was confirmation of the total ablation zone with 5 mm safety margins.

### 2.3. Transcatheter Arterial Chemoembolization

The TACE procedure has been described in detail [15]. Briefly, TACE was operated by interventional radiologists with more than 10 years of experience. Cisplatin (2 mg/kg) was infused into the tumor-feeding artery using a 1.7–2.4-Fr microcatheter (Progreat, Terumo, Tokyo, Japan), followed by infusion of a 1:1 emulsion of cisplatin in lipiodol (Guerbet, Roissy, France) with a maximum dose of 20 mL. This was followed by embolization with Gelfoam slurry (Upjohn, Kalamazoo, MI, USA) until sufficient segmental arterial flow stasis was achieved. All HCCs were treated in a single TACE session. Patients were monitored overnight for observation of possible postembolization syndrome and other adverse events. Patients were initially followed up by laboratory examinations and CT imaging 1 month after TACE, and subsequently by laboratory examinations and CT/MRI every 2–3 months. A repeat TACE procedure was performed when viable HCCs were detected on follow-up CT/MRI images.

### 2.4. Study Endpoint and Definitions

The primary endpoint of this study was overall survival (OS), defined as the time interval between SR plus IORFA or initial TACE and either death from any cause or the last follow-up. Patients were censored on the day of liver transplantation or data analysis (January 2022) [16]. Secondary study endpoints were progression-free survival (PFS) and major complications. PFS was defined as the time interval between SR plus IORFA or initial TACE and tumor progression, as determined by mRECIST guidelines, or death from any cause [17]. Major complications were defined as those requiring additional management, including a hospital stay beyond the expected postoperative course, elevated level of care, substantial morbidity, or death, as determined by the Society of Interventional Radiology guideline [18]. Portal hypertension was defined as esophageal/gastric varices, ascites, splenomegaly with thrombocytopenia (platelet count < 100,000/mm^3^), and/or noticeable portosystemic shunts [7]. Patients were subgrouped by tumor burden (major/minor), as determined by the up-to-7 criteria [19], which could influence the decision to perform SR plus IORFA or TACE.

### 2.5. Statistical Analysis

Differences in patient characteristics were compared with the chi-squared test or Fisher’s exact test. PFS and OS rates were calculated using the Kaplan–Meier method. Factors associated with PFS and OS were analyzed by univariable and multivariable Cox proportional hazards modeling with the backward elimination method. Factors with univariable *p*-values < 0.1 were included in the multivariable analysis. Propensity score, with SR plus IORFA as the dependent variable, was estimated by multiple logistic regression analysis. The full nonparsimonious model included age; sex; etiology of HCC; maximum tumor size; tumor number; tumor extent; presence of portal hypertension; and serum concentrations of bilirubin, albumin, and alpha-fetoprotein (AFP) as independent variables, as well as the interaction terms between variables. Model discrimination was assessed with c statistics (= 0.718), and model calibration was assessed with Hosmer–Lemeshow statistics (chi-squared = 7.748, DF = 8, *p* = 0.459). Patients who underwent SR plus IORFA and TACE were subjected to a maximum 1:3 propensity score matching (PSM) by Greedy matching, with a caliper of 0.2 standard deviations of the logit of the propensity score (Figures S1 and S2) [20]. Balance after matching was determined by calculating absolute standardized differences, with all absolute standardized differences after matching being <0.15. The propensity score-matched set was assessed by Cox proportional hazard models, with robust standard errors that accounted for the clustering of matched pairs. Variables with *p* < 0.1 on univariable analyses were considered covariates in multivariable analysis. All statistical analyses were performed using SAS version 9.3 (SAS Institute, Cary, NC, USA), with two-sided *p*-values < 0.05 considered statistically significant.

## 3. Results

### 3.1. Patient Characteristics

**Figure 1:**
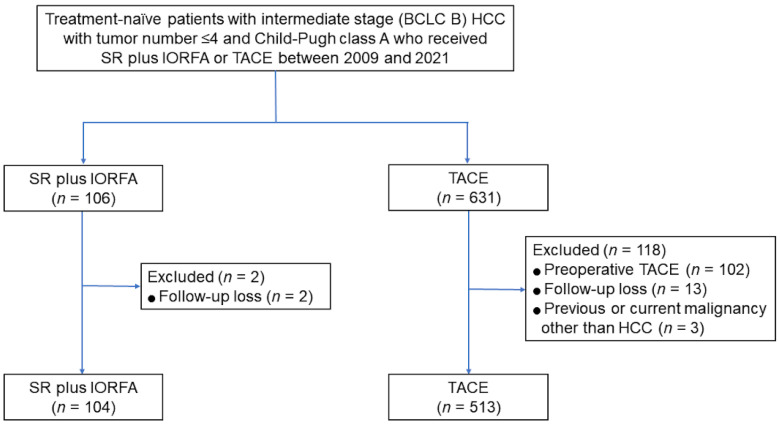
Flow diagram of the study population.

Between January 2009 and December 2021, 106 patients underwent SR plus IORFA, and 631 patients who were potential candidates for SR plus IORFA underwent TACE as first-line treatment; all had intermediate (BCLC B) HCC with ≤4 tumors and Child–Pugh class A. Of these patients, 104 who underwent SR plus IORFA and 513 who underwent TACE met the eligibility criteria and were included in this study (Figure 1). Before PSM, the percentages of male patients were higher in both groups, but the proportion of patients aged > 60 years was lower in the SR plus IORFA than in the TACE group (41.3 vs. 51.7%; SMD = 0.208). Hepatitis B virus infection was the predominant etiology of chronic liver disease in both groups. The proportion of patients with maximal tumor size > 5 cm did not differ significantly in the two groups, whereas the proportion of patients with >2 tumors was lower (33.7 vs. 46.4%; SMD = 0.262), and the proportion of patients with bilobar tumors was higher (62.5 vs. 40.9%; SMD = 0.442) in the SR plus IORFA group than in the TACE group. The proportions of patients with serum bilirubin > 0.9 mg/dL and AFP ≥ 200 ng/mL did not differ significantly in the two groups, whereas the proportions of patients with serum albumin concentrations ≤ 3.5 mg/dL (18.3 vs. 33.1%; SMD = 0.345) and with portal hypertension (8.7 vs. 20.3%; SMD = 0.335) were lower in the SR plus IORFA group than in the TACE group.

The baseline characteristics of patients were more balanced after than before PSM, with no statistically significant differences observed between the two groups in age, tumor number, tumor extent, albumin level, and presence of portal hypertension. Table 1 shows the baseline demographic and clinical characteristics of the patient population before and after PSM.

### 3.2. Overall Survival

**Figure 2:**
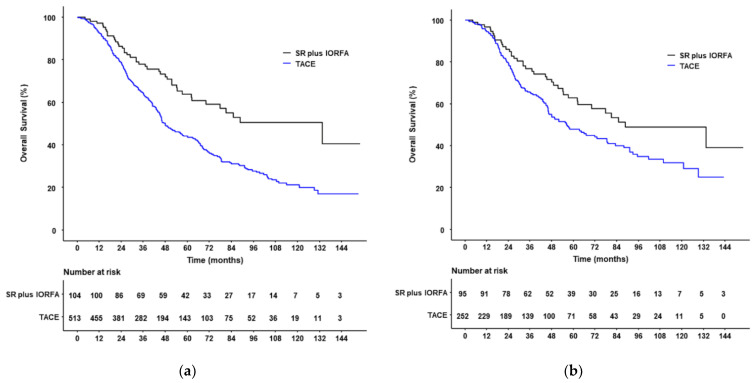
Kaplan–Meier analyses of overall survival (OS) in patients who underwent SR plus IORFA or TACE before (**a**) and after (**b**) PSM. (**a**,**b**) OS rates were significantly higher in patients who underwent SR plus IORFA than in those who underwent TACE both (**a**) before PSM (HR: 0.52 (95% CI, 0.37–0.71), *p* < 0.001) and (**b**) after PSM (HR: 0.65 (95% CI, 0.44–0.95), *p* = 0.026).

At the end of follow-up, before PSM, 41 patients (39.4%) in the SR plus IORFA group and 309 patients (60.2%) in the TACE group had died. The 1-, 3-, 5-, and 10-year cumulative OS rates were 97.1, 77.9, 63.8, and 50.6%, respectively, in the SR plus IORFA group, and 92.4, 64.1, 43.6, and 21.3%, respectively, in the TACE group, indicating that OS rates were significantly higher in patients who underwent SR plus IORFA (HR: 0.52 (95% CI, 0.37–0.71), *p* < 0.001). Median OS was also significantly longer in the SR plus IORFA group than in the TACE group (133.0 months (95% CI, 70.1–NA months) vs. 48.0 months (95% CI, 45.0–57.0 months), *p* < 0.001; Figure 2).

**Figure 3:**
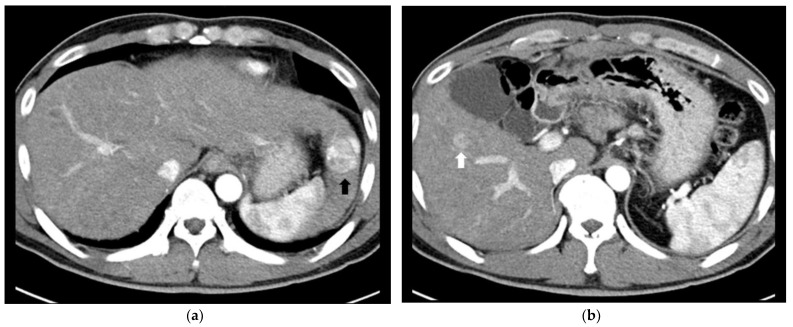

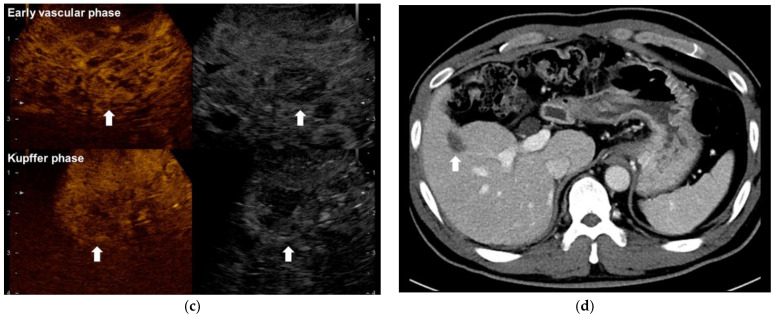
Images of a 39-year-old male patient with intermediate-stage HCC who underwent left lateral sectionectomy plus IORFA. (**a**,**b**) Contrast-enhanced CT images in the arterial phase, showing (**a**) a 3.6 cm HCC in liver segment 2 (black arrow), which was resected, and (**b**) a 1.2 cm HCC in liver segment 5 (white arrow), which could not be resected due to insufficient future liver remnant. (**c**) Sonazoid-enhanced US images during IORFA showing arterial enhancement with a Kupffer defect in liver segment 5 (white arrows). (**d**) CT image 8 years later showing no evidence of a viable HCC in the remnant liver with an RFA defect (white arrow).

After PSM, 39 patients (41.1%) in the SR plus IORFA group and 133 patients (52.8%) in the TACE group had died. The 1-, 3-, 5-, and 10-year cumulative OS rates were 96.8, 76.8, 62.9, and 48.9%, respectively, in the SR plus IORFA group (Figure 3), and 94.3, 65.3, 47.9, and 32.0%, respectively, in the TACE group, indicating that OS rates were significantly higher in PSM patients who underwent SR plus IORFA (HR: 0.65 (95% CI, 0.44–0.95), *p* = 0.026). Median OS was also significantly longer in PSM patients in the SR plus IORFA group than in the TACE group (88.6 months (95% CI, 62.3–NA months) vs. 56.2 months (95% CI, 46.1–78.5 months), *p* = 0.026; Figure 2).

### 3.3. Progression-Free Survival

**Figure 4:**
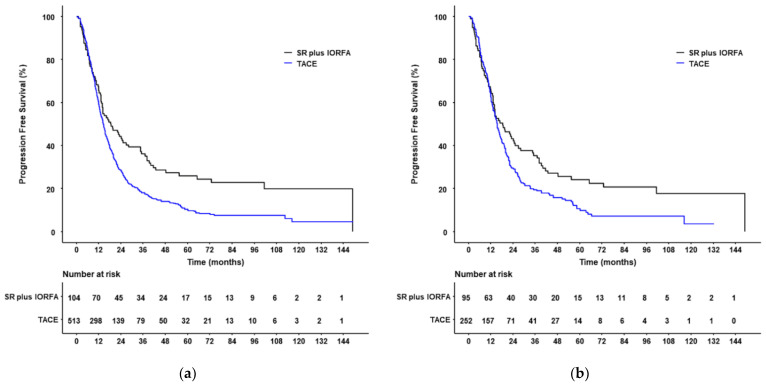
Kaplan–Meier analyses of progression-free survival (PFS) in patients who underwent SR plus IORFA or TACE before (**a**) and after (**b**) PSM. (**a**,**b**) PFS rates were significantly higher in patients who underwent SR plus IORFA group than in those who underwent TACE both (**a**) before PSM (HR: 0.67 (95% CI, 0.52–0.85), *p* < 0.001) and (**b**) after PSM (HR: 0.72 (95% CI, 0.54–0.96), *p* = 0.023).

Before PSM, 79 patients (76.0%) in the SR plus IORFA group and 442 (86.2%) in the TACE group showed HCC progression or died. The 1-, 3-, 5-, and 10-year cumulative PFS rates were 65.4, 36.1, 25.9, and 19.9%, respectively, in the SR plus IORFA group, and 59.1, 18.1, 9.8, and 4.6%, respectively, in the TACE group, indicating that PFS rates were significantly higher in patients who underwent SR plus IORFA (HR: 0.67 (95% CI, 0.52–0.85), *p* < 0.001). Median PFS was also significantly longer in the SR plus IORFA than in the TACE group (18.5 months (95% CI, 14.0–28.2 months) vs. 14.5 months (95% CI, 13.3–15.5 months), *p* < 0.001; Figure 4).

After PSM, 74 patients (77.9%) in the SR plus IORFA group and 216 (85.7%) in the TACE group experienced HCC progression or died. The 1-, 3-, 5-, and 10-year cumulative PFS rates were 65.3, 35.3, 24.1, and 17.8%, respectively, in the SR plus IORFA group, and 62.4, 19.5, 9.8, and 3.6%, respectively, in the TACE group, indicating that PFS rates were significantly higher in patients who underwent SR plus IORFA (HR: 0.72 (95% CI, 0.54–0.96), *p* = 0.023). Median PFS was also significantly longer in the SR plus IORFA group than in the TACE group (18.4 months (95% CI, 13.5–26.8 months) vs. 15.3 months (95% CI, 13.8–17.4 months), *p* = 0.023; Figure 4).

### 3.4. Multivariable Analyses

After PSM, treatment type was significantly associated with both OS and PFS by stratified Cox regression and doubly robust estimation adjusted for factors that were significant in univariate analyses (Table 2). The covariates adjusted for PFS were maximal tumor size, tumor number, and bilirubin and albumin concentrations, whereas the covariates adjusted for OS were maximal tumor size; tumor number; sex; bilirubin, albumin, and AFP concentrations; and portal hypertension.

### 3.5. Subgroup Analyses of OS

**Figure 5:**
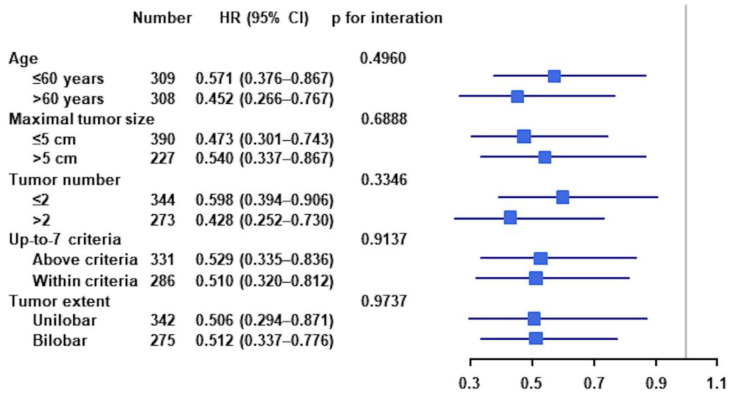
Subgroup analyses of overall survival (OS) in the entire study population.

Patients were subgrouped by age (≤60 vs. >60 years), maximal tumor size (≤5 vs. >5 cm), tumor numbers (≤2 vs. >2), up-to-7 criteria (above vs. within), and tumor extent (unilobar vs. bilobar) to determine the effects of these factors on the improved OS in patients who underwent SR plus IORFA compared with TACE. OS was superior in all subgroups of patients who underwent SR plus IORFA to those who underwent TACE group (Figure 5).

### 3.6. Major Complications

Prior to PSM, major complications occurred in 7 (6.7%) of the 104 patients in the SR plus IORFA group and 33 (6.4%) of the 513 patients in the TACE group (*p* > 0.999). The major complications in the SR plus IORFA group were pleural effusion in five patients and intraabdominal fluid collection in two, requiring catheter drainage procedures. The major complications in the TACE group included allergic reaction related to cisplatin in nine patients; fever in six; acute liver failure, acute renal failure, sepsis, liver abscess, and ischemic cholangiopathy in three patients each; biloma in two; and paralytic ileus in one. After PSM, major complications occurred in 7 (7.4%) of the 95 patients in the SR plus IORFA group, and in 21 (8.3%) of the 252 patients in the TACE group (*p* = 0.942).

## 4. Discussion

To our knowledge, this is the largest study of a PSM population to compare SR plus IORFA with TACE in the treatment of intermediate-stage (BCLC B) HCC with long-term follow-up. During a median follow-up of 41.8 months, both PFS and OS were superior in the SR plus IORFA than in the TACE group. After PSM, both median PFS and OS were significantly longer in the SR plus IORFA than in the TACE group. The cumulative 1-, 3-, 5-, and 10-year OS rates were 96.8, 76.8, 62.9, and 48.9%, respectively, in the SR plus IORFA group, and 94.3, 65.3, 47.9, and 32.0%, respectively, in the TACE group (HR: 0.65, *p* = 0.026). Cox proportional hazard models and doubly robust estimation also found that treatment type was independently associated with both PFS and OS. Major complication rates were comparable in the two groups. These findings may have important implications for the treatment of patients with intermediate-stage (BCLC B) HCC, as in previous studies, which showed the limitation of the BCLC system to guide treatment options and showed better outcomes with more aggressive treatments [5]; no randomized controlled trials (RCTs) to date have compared these treatment modalities.

RFA combined with surgery can be performed either by a pre- or postoperative percutaneous method or by simultaneous IORFA. The latter, however, has several advantages compared with nonsimultaneous percutaneous RFA. First, percutaneous RFA has many limitations in terms of tumor location, with a high risk of complications and low therapeutic effect when an HCC is located adjacent to organs, such as the stomach, large bowel, and gallbladder, or when an HCC is located in a subcapsular position or in a deep location such as a caudate lobe. In contrast, liver mobilization during surgery can create a safe route for major vessels and adjacent organs, or can access an otherwise deeply located caudate lobe, thus increasing technical success rates [21]. Second, direct application of high-resolution US via the liver surface can make it easier to detect HCCs that are otherwise difficult to distinguish from other cirrhotic nodules, with or without the aid of Sonazoid, thus allowing real-time monitoring of RFA throughout the entire procedure end [22,23]. Third, because IORFA is performed under general anesthesia, patients do not experience pain, operators have no restrictions due to patient intolerance, and the procedure can be performed during a single session without the need for additional sedation. Finally, IORFA has fewer limitations associated with the numbers and sizes of tumors than percutaneous RFA due to easy insertion of the electrode at different angles to provide adequate margins on all sides of an HCC [23].

Several single-arm retrospective studies have reported long-term outcomes of SR plus IORFA in patients with multifocal HCC. One study reported that the cumulative 3-, 5- and 7-year OS rates were 84.3, 61.2, and 61.2%, respectively [24], whereas a second study reported cumulative 1-, 3-, and 5-year OS rates of 87, 80, and 55%, respectively [25]. In addition, several studies have compared SR plus IORFA with SR alone in patients with multifocal HCC, with the two groups having comparable OS outcomes [26,27,28]. However, SR plus IORFA in patients with intermediate-stage (BCLC B) HCC should be compared with TACE, as the latter is the recommended first-line therapy and the most widely used treatment option in these patients. Several retrospective studies comparing SR plus IORFA with TACE showed better survival outcomes in patients who underwent SR plus IORFA [11,13]. Those studies, however, had selection biases, with patient characteristics differing in the two groups. Specifically, patients in the TACE group had poorer hepatic function, larger sized tumors, and more tumors than patients in the SR plus IORFA group.

Several studies have compared SR plus IORFA with TACE in PSM patients with multifocal HCC [12,29]. For example, a comparison of 26 patients who underwent SR plus IORFA and 153 who underwent TACE found that OS rates were significantly higher (*p* = 0.011), and time to progression significantly longer (*p* < 0.001), in the SR plus IORFA group [12]. Both univariate and multivariate analyses of the PSM population found that combined therapy was significantly associated with OS and time to progression. A comparison of 59 patients who underwent SR plus IORFA and 410 who underwent TACE found that the 1-, 2-, and 3-year cumulative OS rates were 81.8, 68.7, and 63.4%, respectively, in the SR plus IORFA group, and 59.3, 36.1, and 19.4%, respectively, in the TACE group, after PSM (*p* < 0.001) [29]. Subgroup analysis found that SR plus IORFA was associated with better OS outcomes in all subgroups. Those studies, however, included smaller patient populations and did not report long-term outcomes. In addition, although those studies reported p-values, they did not report standardized mean differences before and after PSM, a more appropriate comparison when analyzing propensity-matched population status.

This study is different from others since it was performed with a relatively large sample size (*n* = 104 vs. *n* = 513). Moreover, the median follow-up period (41.8 months) in our study was relatively longer than those of other studies (27.0–35.7 months) [11,13,29], which allowed the calculation of cumulative OS rates up to 10 years. Lastly, we presented standardized mean difference of patient’s characteristics before and after PSM and were able to show the balanced status of PSM and minimize potential confounders.

The 2022 update of BCLC treatment recommendations classified BCLC B into three categories, one of which corresponds to liver transplant candidates who meet extended liver transplant criteria [30]. Similarly, the present study showed that SR plus IORFA provided better survival outcomes than TACE in BCLC B patients who were candidates for SR plus IORFA. SR plus IORFA may therefore play an important role in selected patients with BCLC B HCC.

Locoregional treatments for HCC has continued to evolve over the years. New-generation multipolar RFA and multiprobe microwave ablation can obtain larger ablation zones, and other ablative methods, such as cryoablation or irreversible electroporation, are alternatives and under investigation. In addition, several techniques have been developed to improve the efficacy of TACE such as drug-eluting bead TACE. Radioembolization has also continued to develop, such as boosted radioembolization (ablative radioembolization or radiation segmentectomy; >190 Gy), which shows curative potential and been used as a strategy to enable resection or transplantation [31].

The emergence of immune checkpoint inhibitors (ICI) has rapidly opened new opportunities and expanded the treatment strategies of HCC, and not only in the advanced stage [32]. Locoregional treatments can escalate tumor immunogenicity by activating a pro-immune inflammatory response and releasing tumor-associated antigens, which can boost systemic anticancer immune responses. In addition, baseline immune status (high neutrophil-to-lymphocyte ratio) was found to be a significant predictor of OS after TACE [33], thus providing a solid rationale for the combination of locoregional treatments with ICI. Due to these factors, ICI have been thought to be beneficial in the adjuvant or neoadjuvant setting for patients with high risk of recurrence after complete resection or ablation. Currently, many RCTs are investigating ICI-based combinations, and their results are eagerly awaited [32,34,35].

The present study has several limitations. First, its retrospective design may have introduced potential selection bias. Even after PSM, some confounding factors may have been present. Second, this study was performed on patients treated at a single tertiary center in the Asia–Pacific region. External validation may be needed for patients in other countries, owing to differences in demographic characteristics and underlying etiologies of liver diseases. Randomized controlled, multicenter trials are therefore warranted.

## 5. Conclusions

In conclusion, SR plus IORFA showed better OS and PFS outcomes than TACE in patients with intermediate-stage (BCLC B) HCC. SR plus IORFA should be considered as the preferred treatment option for eligible patients whenever possible. SR plus IORFA may provide curative treatment to patients with intermediate-stage HCC who are otherwise deemed eligible only for palliative treatment.

## Figures and Tables

**Table 1:** Demographic and clinical characteristics of patients before and after propensity score matching.

Variables	Before PSM			After PSM		
	SR plus IORFA	TACE	SMD	SR plus IORFA	TACE	SMD
No. of patients	104	513		95	252	
Age > 60 years, *n* (%)	43 (41.3%)	265 (51.7%)	0.208	40 (42.1%)	112 (44.4%)	0.047
Male sex, *n* (%)	90 (86.5%)	443 (86.4%)	0.005	81 (85.3%)	226 (89.7%)	0.134
Etiology			0.143			0.067
HBV	74 (71.2%)	373 (72.7%)		70 (73.7%)	191 (75.8%)	
HCV	8 (7.7%)	61 (11.9%)		8 (8.4%)	21 (8.3%)	
Alcohol	8 (7.7%)	46 (9.0%)		8 (8.4%)	17 (6.7%)	
Others	14 (13.5%)	33 (6.4%)		9 (9.5%)	23 (9.1%)	
Maximal tumor size > 5 cm, *n* (%)	44 (42.3%)	183 (35.7%)	0.136	38 (40.0%)	98 (38.9%)	0.023
Tumor number > 2, *n* (%)	35 (33.7%)	238 (46.4%)	0.262	33 (34.7%)	101 (40.1%)	0.111
Bilobar tumor extent, *n* (%)	65 (62.5%)	210 (40.9%)	0.442	56 (58.9%)	133 (52.8%)	0.124
Bilirubin > 0.9 mg/dL, *n* (%)	25 (24.0%)	154 (30.0%)	0.135	25 (26.3%)	66 (26.2%)	0.003
Albumin ≤ 3.5 mg/dL, *n* (%)	19 (18.3%)	170 (33.1%)	0.345	19 (20.0%)	55 (21.8%)	0.045
Portal hypertension, *n* (%)	9 (8.7%)	104 (20.3%)	0.335	9 (9.5%)	32 (12.7%)	0.103
AFP ≥ 200 ng/mL, *n* (%)	25 (24.0%)	155 (30.2%)	0.139	25 (26.3%)	54 (21.4%)	0.115

Abbreviations: HBV, hepatitis B virus; HCV, hepatitis C virus; AFP, alpha-fetoprotein; PSM, propensity score matching; SR, surgical resection; IORFA, intraoperative radiofrequency ablation; TACE, transcatheter arterial chemoembolization; SMD, standardized mean difference.

**Table 2:** Cox proportional hazards analyses of SR plus IORFA versus TACE on PFS and OS.

Analyses	HR	95% CI		*p*-Value
PFS				
Unadjusted	0.665	0.523	0.847	0.0009
Adjusted	0.675	0.529	0.86	0.0015
Propensity score matched *	0.718	0.539	0.956	0.0234
Propensity score matched and adjusted for selected variables ^†^	0.695	0.517	0.933	0.0155
OS				
Unadjusted	0.515	0.371	0.713	<0.0001
Adjusted	0.542	0.39	0.752	0.0003
Propensity score matched *	0.647	0.441	0.95	0.0263
Propensity score matched and adjusted for selected variables ^‡^	0.579	0.384	0.874	0.0092

HR, hazard ratio; CI, confidence interval; PFS, progression-free survival; OS, overall survival. * Cox proportional hazard models, with robust standard errors that accounted for the clustering of matched pairs. ^†^ Adjusted for maximal tumor size, tumor number, and bilirubin and albumin concentrations, all of which were significant on univariable analyses. ^‡^ Adjusted for maximal tumor size; tumor number; sex; bilirubin, albumin, and AFP concentrations; and portal hypertension, all of which were significant on univariable analyses.

## Data Availability

The data presented in this study are available on request from the corresponding author.

## Supplementary Materials

The following are available online at https://www.mdpi.com/article/10.3390/cancers14102440/s1, Figure S1: A Jitter plot of propensity score distribution before and after matching. Figure S2: Histograms of propensity score distribution before and after matching.
