# Supplementary material for: Surgical Resection plus Intraoperative Radiofrequency Ablation versus Chemoembolization for the Treatment of Intermediate-Stage (BCLC B) Hepatocellular Carcinoma with Preserved Liver Function: A Propensity Score-Matched Analysis
Source: Cancers (Basel). 2022 May 15;14(10):2440. doi: 10.3390/cancers14102440 (PMC9139238; doi:10.3390/cancers14102440)
Supplement: Supplementary file 1 [file cancers-14-02440-s001.zip › cancers-1725522-supplementary.pdf]

cancers

Supplementary Materials

Surgical Resection plus Intraoperative Radiofrequency Ablation versus Chemoembolization for the Treatment of Intermediate-Stage (BCLC B) Hepatocellular Carcinoma with Preserved Liver Function: A Propensity Score-Matched Analysis

Gun Ha Kim, Jin Hyoung Kim, Heung Kyu Ko, Hee Ho Chu, Seong Ho Kim, Ji Hoon Shin, Dong Il Gwon, Gi-Young Ko, Hyun-Ki Yoon, Ki-Hun Kim, Ju Hyun Shim and Nayoung Kim

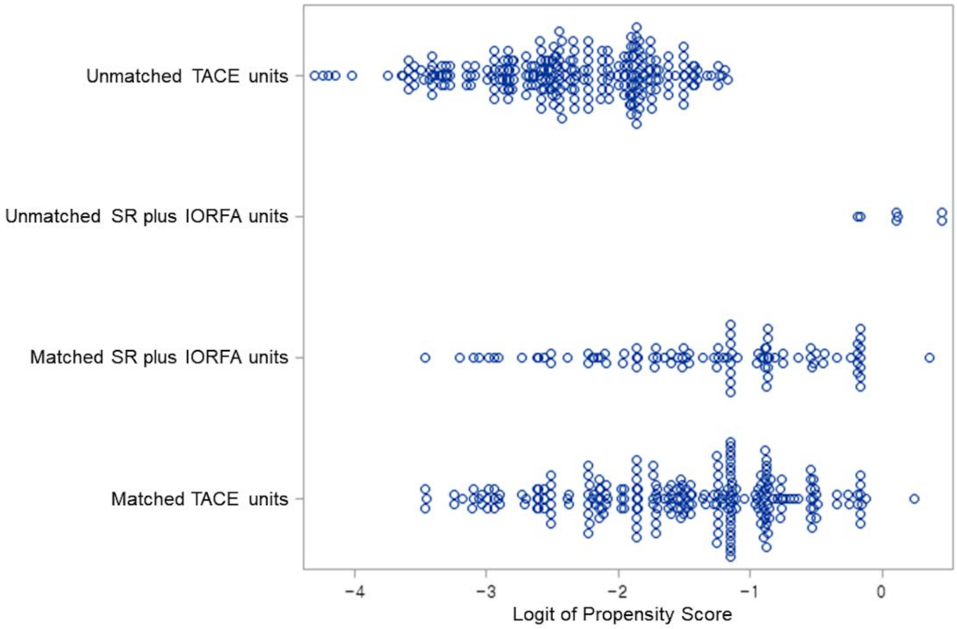

Figure S1. A Jitter plot of propensity score distribution before and after matching.

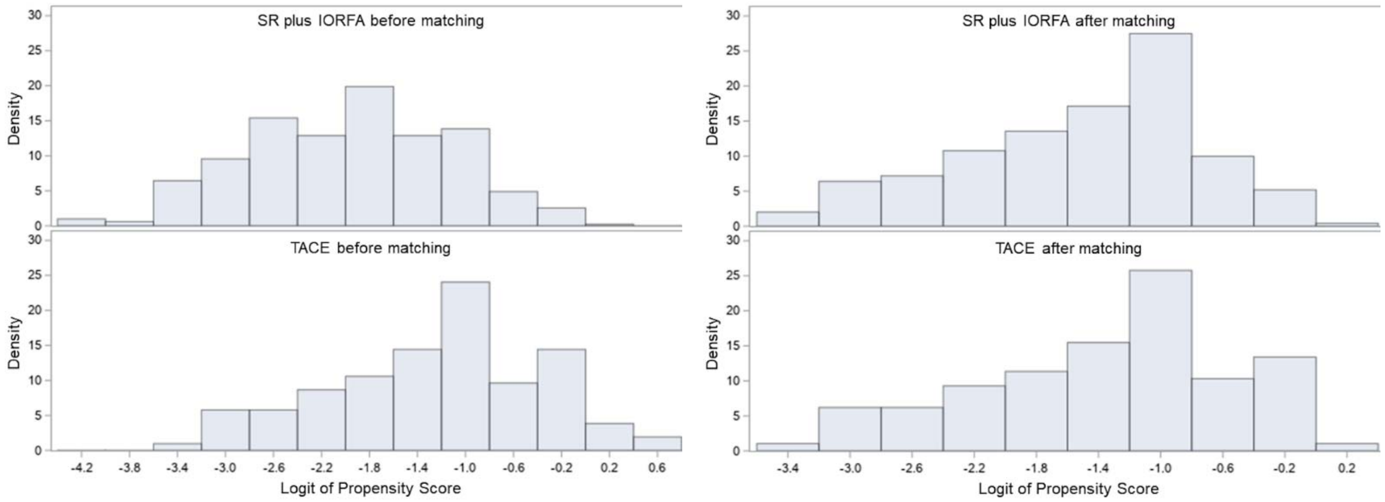

(**a**) (**b**)

Figure S2. Histograms of propensity score distribution before (**a**) and after (**b**) matching.
